# Assessment of the efficacy of different procedures that remove and disassemble alpha-synuclein, tau and A-beta fibrils from laboratory material and surfaces

**DOI:** 10.1038/s41598-018-28856-2

**Published:** 2018-07-17

**Authors:** Alexis Fenyi, Audrey Coens, Tracy Bellande, Ronald Melki, Luc Bousset

**Affiliations:** Paris-Saclay Institute of Neuroscience, Centre National de la Recherche Scientifique, Université Paris-Saclay, 91190 Gif-sur-Yvette, France

## Abstract

α-synuclein fibrillar polymorphs, Tau and Aß 1–42 fibrillar assemblies have been shown to propagate, amplify and trigger the formation of protein deposits reminiscent of those present within the central nervous system of patients developing synucleinopathies, tauopathies and amyloid plaques after injection intracerebrally, intramuscularly, intraperitoneally or within the blood stream of model animals. They are thus hazardous and there is need for decontamination and inactivation procedures for laboratory surfaces and non-disposable material. We assessed the effectiveness of different reagents to clean and disassemble potentially pathogenic assemblies adsorbed on non-disposable materials in laboratories. We show that commercial detergents and SDS are way more suited to detach α-synuclein fibrillar polymorphs, Tau and Aß 1–42 fibrillar assemblies from contaminated surfaces and disassemble the fibrils than methods designed to decrease PrP prion infectivity. Our observations reveal that the choice of the most adapted cleaning procedure for one given protein assembly or fibrillar polymorph should integrate detergent’s cleaning efficiency, material compatibility and capacity to dismantle assemblies. We provide an integrated representation where desorption and neutralization efficacy and surface compatibility are combined to facilitate the choice of the most adapted decontamination procedure. This representation, together with good laboratory practices, contributes to reducing potential health hazards associated to manipulating protein assemblies with prion-like properties.

## Introduction

The proteins alpha-synuclein (α-Syn), Tau and the amyloid peptide Aß 1–42 are the principal component of Lewy bodies and neurites, tangles and Paired Helical Filaments (PHF) and amyloid plaques, respectively. These microscopic protein particles are the neuropathological hallmarks of Parkinson’s disease and other synucleinopathies and Alzheimer’s disease and other tauopathies^1^. The monomeric forms of α-Syn, Tau and Aß1–42 form *in vitro* under physiological pH and salt conditions a continuum of oligomeric assemblies ranging from low to high molecular weight protein particles, the largest of which have fibrillar shape^2^. These assemblies, have been shown to propagate, amplify and trigger the formation of protein deposits reminiscent of those present within the central nervous system of patients developing synucleinopathies, tauopathies and amyloid plaques after their take up by cultured cells and/or upon injection within the central nervous system of model animals^3–34^. α-Syn, Tau and Aß 1–42 assemblies were also shown to reach the CNS by crossing for instance the brain blood barrier when injected systemically, intraperitoneally or intramuscularly into model animals^20,30^. These observations suggest that α-Syn, Tau and Aß 1–42 assemblies may contaminate the CNS from peripheral tissues. Thus, α-Syn, Tau and Aß 1–42 assemblies need to be considered as potentially hazardous material within the laboratory. Consequently, to minimize the risk associated to exposure of workers in the laboratory to those assemblies it is not only important to develop guidelines for how to best handle those assemblies in the laboratory but also to develop cleaning methods that allow reducing the potential hazard associated to their manipulation.

Decontamination and inactivation procedures allowing health hazard reduction by a factor 1000 to 10000 have been implemented in the prion field for reusable material. These procedures are based on the use of sodium hypochlorite (20,000 ppm available free chlorine), sodium hydroxide (1 N) and autoclaves (121–134 °C for 1 hour) or a combination of hydrogen peroxide and copper ions^35–37^. We recently showed that the procedures recommended for reducing the infectivity of prion protein particles are not best suited to clean material contaminated by α-Syn assemblies^38^. This is certainly due to the fact that protein particles, made of different proteins, have distinct properties. We therefore assessed the ability of a variety of cleaning procedures, including practices established for the infectious form of prion protein, to remove α-Syn, Tau and Aß 1–42 assemblies adsorbed on plastic, glass, aluminum or stainless steel surfaces that equip and/or are most commonly used in laboratories. We also evaluated in a quantitative manner the protein assemblies disassembling propensities of those cleaning procedures.

We show here that the cleaning procedure efficiency depends not only on the nature of the protein assembly but also on the structural characteristics of distinct assemblies made of one given protein e.g. polymorphs. Our results strongly suggest that the cleaning procedure needs being adapted to the nature of the contaminated surface. Assessment of the fate of the distinct assemblies and polymorphs that were detached from contaminated surfaces shows that the most efficient cleaning procedures do not necessarily disassemble the assemblies. Thus, the choice of the most adapted cleaning procedure for one given protein assembly or polymorph should integrate the procedure cleaning efficiency and capacity to dismantle assemblies.

## Results

### Binding of α-Syn, Tau and Aß fibrillar assemblies

**Figure 1:**
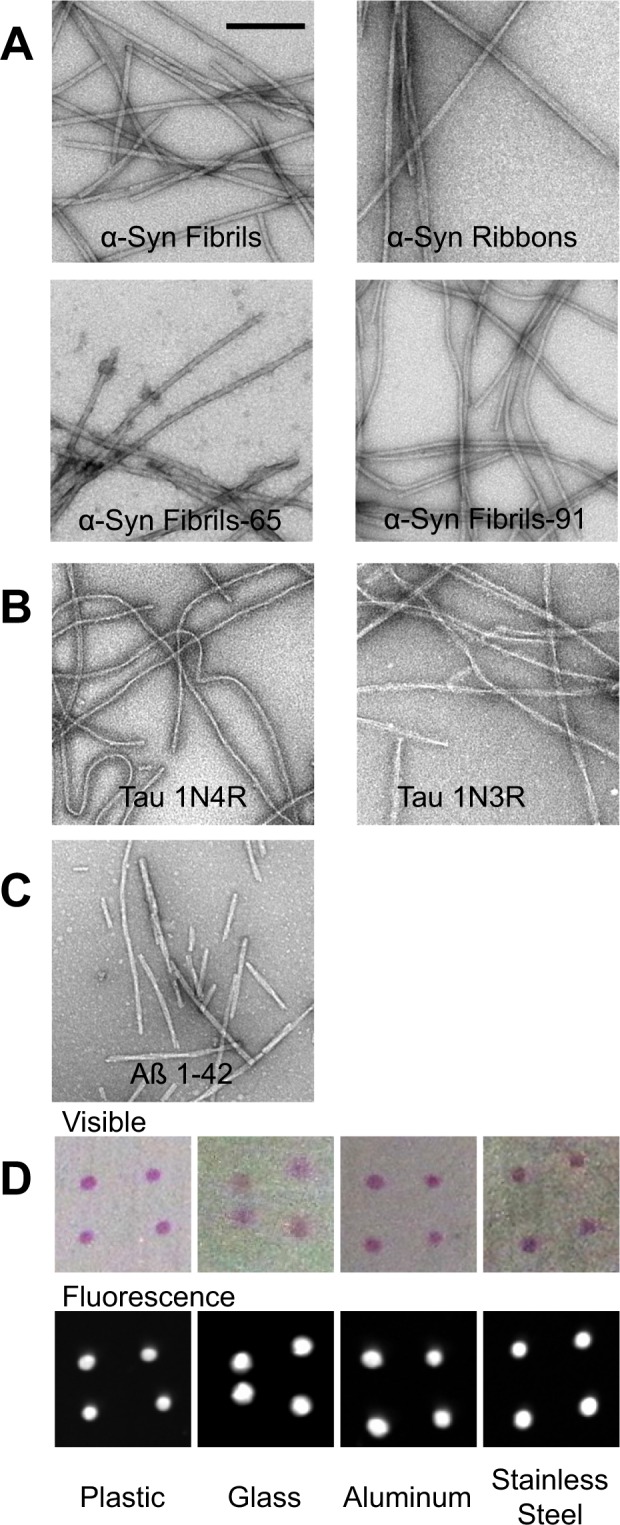
Fibrillar assemblies and experimental setup (**A**–**C**) electron micrographs of fibrillar assemblies. α-Syn fibrillar polymorphs (**A**), Tau fibrillar assemblies 1N3R or 1N4R (**B**), and Aß1–42 peptide fibrils (**C**). Representative images of the different plates used throughout the present study after spotting and drying 4 droplets of α-Syn fibrils labeled with Atto 550 imaged in visible light and by fluorescence (**D**). Scale bar, 200 nm.

Distinct α-Syn fibrillar polymorphs, also designated strains, obtained under well-defined assembly conditions (Fig. 1A), Tau fibrils made of Tau 1N3R and 1N4R isotypes (Fig. 1B) and Aß1–42 fibrils (Fig. 1C) were generated, labeled with Atto-550 succinimidyl ester and spotted on strips made of plastic, glass, stainless steel, or aluminum. To better mimic the conditions encountered in laboratories where the surfaces are not necessarily smooth, all surfaces were pre-treated with sandpaper to make the surface rougher, increase the attachment of the material and diminish elimination by gentle wiping. To further mimic what may happen in a laboratory after surfaces contamination, droplets containing α-Syn, Tau and Aß fibrillar assemblies were allowed to dry overnight at room temperature. The different fibrillar assemblies adhered to the distinct surfaces and the spots were clearly visible by eye or by fluorescence (Fig. 1D).

### Cleaning procedure

**Figure 2:**
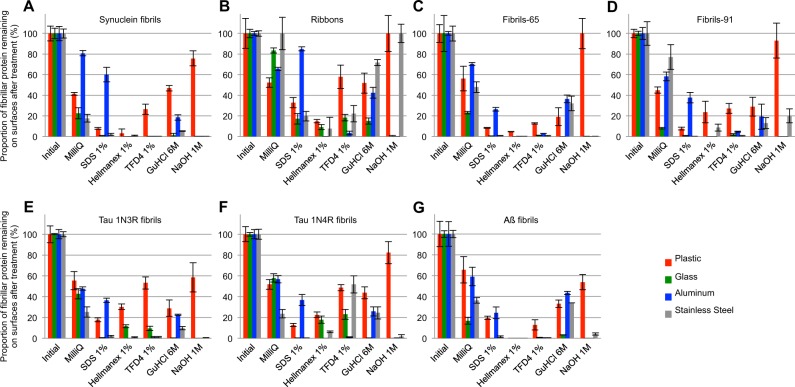
Efficiency of different cleaning solutions to remove amyloid fibrillar assemblies from plastic, glass, aluminum and stainless steel surfaces. Quantification of the remaining Atto-550 labeled fibrillar α-Syn polymorph “fibrils” (**A**), “ribbons” (**B**), “fibrils-65” (**C**), “fibrils-91” (**D**), Tau 1N3R fibrils (**E**), Tau 1N4R fibrils (**F**) and Aß fibrils (**G**) spotted and dried on plastic (red), glass (green), aluminum (blue) and stainless steel (grey) surfaces, previously scraped with sandpaper, using a fluorescence imager after cleaning with the different solutions. Error bars represent standard error (SE) (n = 4 independent measurements) performed in quadruplicates.

**Figure 3:**
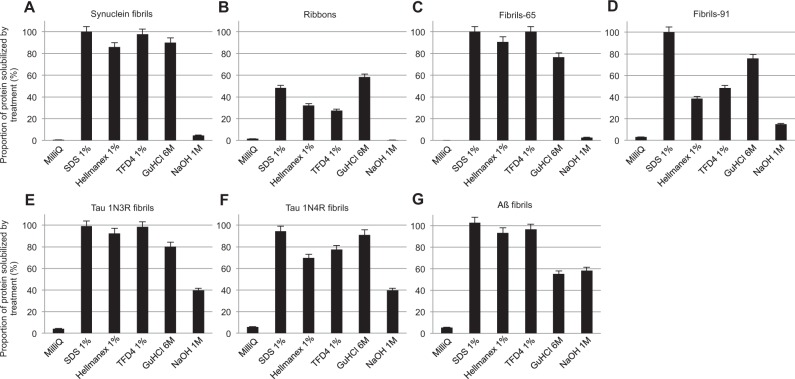
Quantitative assessment of the fraction of non-fibrillar Atto-550 labeled α-Syn, Tau and Aß in the different cleaning solutions. The fraction (%) of the α-Syn fibrillar polymorphs “fibrils” (**A**), “ribbons” (**B**), “fibrils-65” (**C**), “fibrils-91” (**D**), Tau 1N3R fibrils (**E**), Tau 1N4R fibrils (**F**) and Aß fibrils (**G**), that becomes soluble in the cleaning solution was determined following ultracentrifugation by measurement of the absorbance of fluorescently labeled protein in the supernatant fraction as described in the material and methods section. Error bars represent standard error (SE) (n = 4 independent measurements) performed in quadruplicates.

The washing procedure consisted of immersing the contaminated surfaces in washing solutions of different compositions under gentle agitation on an orbital shaker as described in the methods section. The surfaces were washed with MilliQ water, dried and the amount of assemblies that remained after washing (Fig. 2) was quantified by fluorescence measurements. The cleaning solution may disassemble the fibrillar assemblies detached from the surfaces to different extents. In the case no disassembly occurs within the cleaning solution, the latter needs to be handled as a biohazard while no such requirement is needed in the case of complete disassembly. We therefore assessed the amount of α-Syn fibrillar polymorphs, Tau and Aß fibrillar assemblies remaining after 1 hour treatment in the cleaning solutions following centrifugation (Fig. 3).

### Quantitative assessment of the cleaning efficacy of different washing solutions

The amount of α-Syn fibrillar polymorphs, Tau and Aß fibrillar assemblies that remained attached to the different surfaces after washing with each one of the following solutions: (i) MilliQ water; (ii) SDS (1%); (iii) Hellmanex II (1%); (iv) TFD4 (1%); (v) guanidinium hydrochloride (6 M) and (vi) NaOH (1 M) was determined by measuring the amount of fluorescent α-Syn fibrillar polymorphs, Tau and Aß fibrillar assemblies remaining on the contaminated surfaces (Fig. 2). The total amount of the different α-Syn fibrillar polymorphs, Tau 1 N3R, 1N4R and Aß fibrils spotted initially on the different surfaces was arbitrarily set to 100%. The amount of α-Syn fibrillar polymorphs, Tau and Aß fibrillar assemblies remaining on the different surfaces after the washing step, represented by the measured fluorescence intensity, was expressed relative to the initial 100% fluorescence intensity of each fibrillar assembly. The washing solutions did not affect the fluorescence of the ATTO-550 dye after the washing step (Figure S1). Fibrillar assemblies made of different proteins were removed by the different cleaning solutions with different efficiencies. Indeed, while the proportion of α-Syn and Aß fibrils remaining on glass surfaces was undetectable upon cleaning with 1% Hellmanex in MilliQ water, that of Tau fibrils was 10 to 20%. Similarly, while the proportion of α-Syn fibrillar polymorph “fibrils” remaining on glass surfaces was undetectable upon cleaning with 1% SDS in MilliQ water, that of the α-Syn fibrillar polymorphs “ribbons” was nearly 20%. Finally, while the proportion of α-Syn fibrillar polymorph “fibrils” remaining on stainless steel surfaces was undetectable upon cleaning with 1% SDS in MilliQ water, that of the same fibrillar polymorph on aluminum surfaces was 60%. We conclude from these measurements that the cleaning solutions need to be adapted not only to the nature of the fibrillar protein assemblies but also to the fibrillar polymorph and the nature of the contaminated material. Similar results were obtained for Tau and Aß fibrils. SDS (1% in MilliQ water) allowed the desorption of over 99% of Tau 1N3R, 1N4R and Aß fibrils from glass, outranging the cleaning efficacy of Hellmanex and TFD4 (1% in MilliQ water), the proportion of fluorescent fibrils remaining on aluminum surfaces after cleaning with SDS was 37 ± 5%. Overall, the commercial detergent Hellmanex (1% in MilliQ water), was most efficient for cleaning all surfaces (Fig. 2). The commercial detergent TFD4 (1% in MilliQ water) and SDS (1% in MilliQ water) performed well except for aluminum surfaces for SDS and plastic surfaces for TFD4. Sodium hydroxide (1N in milliQ water) performed well except for plastic and stainless steel surfaces. It is worth noting that NaOH, Hellmanex and TFD4 corrode aluminum surfaces (Figure S2).

### Quantitative assessment of the fate of α-Syn, Tau and Aß fibrillar polymorphs

To determine the fate of desorbed α-Syn fibrillar polymorphs, Tau and Aß fibrillar assemblies, in particular whether they remain fibrillar or disassemble into low molecular weight species in the washing solutions, the different assemblies were incubated with the cleaning solutions used to clean plastic, glass, steel, or aluminum in this study for 1 h at room temperature and subjected to ultracentrifugation. The amount of non-fibrillar, fluorescent α-Syn, in the supernatant of the different solutions used throughout this study, expressed as the fraction of the initial fibrillar assemblies before centrifugation, was quantified (Fig. 3). All α-Syn fibrillar polymorphs, Tau and Aß fibrillar assemblies remained of fibrillar nature in MilliQ water as assessed by ultracentrifugation. Overall, SDS (1% in MilliQ water), was most efficient for disassembling fibrillar Tau, Aß and α-Syn fibrillar polymorphs. The α-Syn fibrillar polymorph “ribbons” resisted most to SDS-mediated disassembly. The commercial detergents Hellmanex and TFD4 performed relatively well except for α-Syn fibrillar polymorph “ribbons” and “fibrils-91”. Sodium hydroxide (1 N in MilliQ water) did not disassemble efficiently α-Syn fibrillar polymorphs, Tau and Aß fibrillar assemblies. Finally, Guanidine hydrochloride (6 M in MilliQ water) performed best for the α-Syn fibrillar polymorph “ribbons” with 58 ± 3% disassembly. We conclude from these measurements that the cleaning solutions need to be adapted to the nature of the fibrillar protein assemblies and to the fibrillar polymorphs.

### Overall efficacy of the different cleaning strategies

**Figure 4:**
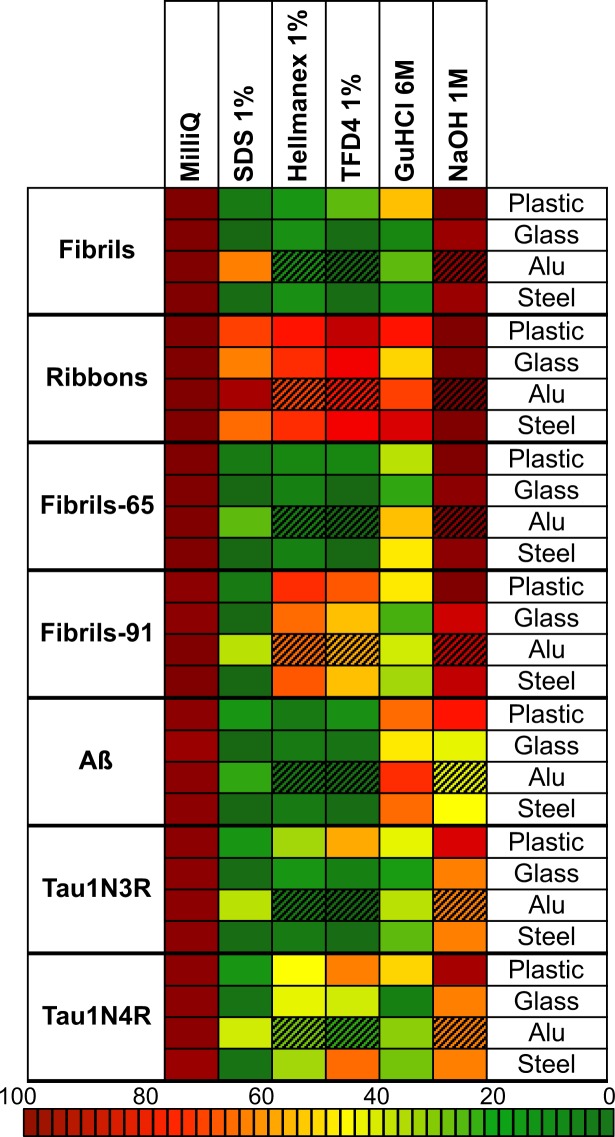
Combined representation of the cleaning procedures desorption and neutralization efficacies and compatibility with the contaminated surfaces. This representation combines the desorption and fibrils disassembly efficiency of each experimental treatment and the compatibility of the treatment with the nature of the surfaces. Colors spanning from brown (high) to dark green (low) integrate the proportion of material remaining adsorbed and of fibrillar nature. Hatched boxes indicate that the treatment corrodes the surface.

Taken together, our measurements demonstrate that SDS, TFD4 and Hellmanex (1% in MilliQ water) are overall the most efficient cleaning procedures as they detach and disassemble α-Syn fibrillar polymorphs, Tau and Aß fibrillar assemblies to the highest extent and to a level where they become undetectable by the method used here (Figs 2 and 3). MilliQ water and sodium hydroxide (1 N in MilliQ water) appear the least efficient cleaning procedures (Figs 2 and 3). As the cleaning solutions need to be adapted to the nature of the fibrillar protein assemblies, the fibrillar polymorphs and to the nature of the contaminated surface, we combined the desorption and neutralization efficacy of each treatment into a unique representation (Fig. 4). This representation allows choosing the most suitable cleaning reagents for removal and neutralization of α-Syn fibrillar polymorphs, Tau and Aß fibrillar assemblies as a function of the contaminated surface nature at a glance (Fig. 4).

## Discussion

None of the fibrillar assemblies listed and used in this work has been demonstrated to be infectious in humans. Nonetheless, combining decontamination and inactivation procedures for reusable material and surfaces with laboratory standard operating procedures for protein assemblies with prion-like properties (Table 1) allows reducing putative health hazards associated to manipulating such protein assemblies in laboratories by a very significant factor exceeding 1000 to 10000 fold. We previously showed that methods designed to decrease PrP prion infectivity that are based on the use of sodium hypochlorite (20,000 ppm available free chlorine), sodium hydroxide (1 N) and autoclaves (121–134 °C for 1 hour) or a combination of hydrogen peroxide and copper ions^35–37^ neither effectively remove a well-defined α-Syn fibrillar polymorphs adsorbed to different materials commonly used in the laboratory nor disassemble it with efficiency. This is certainly due to the fact that protein particles, made of different proteins, have distinct properties. The aim of this work was to expand the previous work and to establish a decontamination and inactivation procedure for several other protein assemblies and fibrillar polymorphs/strains that have been reported to act as seeds in model animals as they trigger the aggregation of endogenous like proteins.

**Table 1:** Laboratory Standard Operating Procedures for fibrillar protein assemblies with prion-like properties.

Recommendations:		
Step	Do	Do NOT
General	Use appropriate personal protective equipment: - Laboratory coat - Gloves - Goggles/safety glasses - Mechanical filter respirators such as FFP2 particulate respirator mask, brand 3 M ref #8822 - Prefer disposable supplies	- Eat or drink in an environment where α-Syn fibrillar strains, Tau and Aß fibrillar assemblies are used
Purification	- Keep the concentration of α-Syn fibrillar strains, Tau and Aß fibrillar assemblies below 1 mM - Maintain pH > 6.5 to avoid spontaneous assembly for α-Syn and < 8 for Tau - Aliquot α-Syn, Tau and Aß upon purification (1 to 5 mg per fraction)	- Concentrate α-Syn, Tau and Aß above 1 mM - Sonicate continuously α-Syn, Tau and Aß solution above 5 min
Fibrillization	- Use the minimal amount of α-Syn fibrillar strains, Tau and Aß fibrillar assemblies needed for the experiment - Assemble in sealed tubes - Sonicate in closed/sealed tubes using appropriated apparatus such VialTweeter or cup-horn sonotrode. - Operate under a PSM2 if working with open tubes/vials	- Sonicate in open containers. This generates aerosol containing α-Syn fibrillar strains, Tau and Aß fibrillar assemblies that conceivably might reach the brain through the olfactory epithelium or the gut following inhalation and swallowing.
Storage	- Keep fibrils in closed tubes and discard in biohazard container immediately after use. - Keep α-Syn fibrillar strains, Tau and Aß fibrillar assemblies in solution	- Do not dry any fibrillar assembly on any surfaces, as this renders them more resistant to detergent solubilization/inactivation.
Inactivation	- Inactivate samples and contaminated surfaces with 1% SDS or commercial inactivation solutions for 1 hours at room temperature - Discard inactivated samples with limited volume (e.g. less than 100 l) in Biohazard waste container	- Do not use MilliQ water, NaOH or Sodium Hypochlorite
Recommendations:		
α-Syn fibrillar strains, Tau and Aß fibrillar assemblies waste	Inactivation	
Fibrillar strains and assemblies in solution	Dilute the solution 10 folds in inactivation solution Incubate 1 h at room temperature Discard in biohazard waste SDS (1% in MilliQ water is best suited for all strains and assemblies except α-Syn ribbons where Guanidine hydrochloride, 6 M, is slightly more efficient)	
Surfaces contaminated with fibrillar strains and assemblies	Immerse completely in inactivation solution Incubate 1 h at room temperature under gentle shaking Rinse with water Discard inactivation solution in biohazard waste If the surface is a bench, wipe with inactivation solution, discard tissues in solid biohazard waste.	

We assessed the effectiveness of different commercially available cleaning solutions to remove and dismantle distinct α-Syn fibrillar polymorphs, Tau and Aß fibrillar assemblies following their adsorption on plastic, glass, aluminum or stainless steel surfaces that are most commonly used in laboratories. The results we report show unequivocally the need to adapt the cleaning procedure to the nature of the fibrillar protein assemblies and the fibrillar polymorphs. Indeed, the cleaning procedure efficiency appears to depend on the nature of the protein assembly and the structural characteristics of distinct assemblies made of one given protein e.g. polymorphs/strains. Our results also reveal that the nature of the contaminated surface strongly influences the cleaning procedure efficacy. Our measurements show that the most efficient desorption procedures do not necessarily dismantle distinct assemblies and polymorphs. Finally, our assessment reveals incompatibilities between certain detergents and surfaces. Thus, the choice of the most adapted cleaning procedure for one given protein assembly or polymorph should integrate detergent’s cleaning efficiency, compatibility with the contaminated surface and capacity to dismantle assemblies.

Overall three cleaning procedures relying on the use of commercially available detergents, SDS, TFD4 and Hellmanex (1% in MilliQ water) effectively removed from non-disposable plastic, glass, aluminum and stainless steel laboratory tools and disassembled distinct α-Syn fibrillar polymorphs, Tau and Aß fibrillar assemblies. While very efficient in removing and disassembling proteins assemblies with prion-like properties, the detergents TFD4 and Hellmanex corroded aluminum surfaces (Figure S2). They thus appear not to be adequate for decontaminating non-disposable laboratory tools made of aluminum. A unique representation (Fig. 4) where different cleaning procedures desorption and neutralization efficacies and compatibility with the contaminated surfaces are combined is given to facilitate the choice of the most suitable and efficient decontaminating procedure. This representation contributes to reducing potential health hazards associated to manipulating protein assemblies with prion-like properties in laboratories.

It is worth noting that cleaning was achieved throughout this study by immersing the contaminated surfaces in washing solutions of different compositions under gentle agitation. Wiping the contaminated surfaces following their exposure to the cleaning solutions with inactivation solution and disposable tissues improves decontamination while generating additional waste.

## Methods

### Reagents used for decontamination

The following reagent were used in this studies: 10% SDS solution (Euromedex # EU0760), Hellmanex II (Hellma #500300.11-F10W), TFD4 (Franklab), NaOH (Euromedex # 21162), MilliQ water (Millipore using a “Milli-Q® Reference”), Guanidine hydrochloride (Sigma, #509501KG).

### Preparation of protein assemblies

#### Expression and purification of protein

The expression and purification of human wild-type α-Syn was performed as previously described^39^. For Tau 1N3R and Tau 1N4R, full-length human Tau1N3R or Tau 1N4R cDNA was cloned in the pET14b vector. hTau (Tau1N3R or Tau 1N4R) was expressed in E. coli BL21 DE3 CodonPlus cells (Stratagene). Cells were grown in LB medium to an optical density at 600 nm of 0.8 absorbance units. hTau expression was induced with 0.5 mM IPTG for 3 h. The cells were then harvested by centrifugation (4000 g, 10 min). The bacterial pellets were resuspended in lysis buffer (20 mM MES pH 6.8, 500 mM NaCl, 1 mM EGTA, 0.2 mM MgCl2, 5 mM di-thiothreitol, 1 mM PMSF + 1 tablet of Complete (Roche)) per liter and lysed by sonication. Cell extracts were clarified by centrifugation at 14,000 g, 30 min. The lysate was heated to 80 °C for 20 min and centrifuged at 14,000 g for 30 min. The supernatant was dialyzed against 100 volumes of buffer A (20 mM MES pH 6.8, 50 mM NaCl, 1 mM EDTA, 1 mM MgCl2, 2 mM DTT, 0.1 mM PMSF) at 4 °C. The dialyzed protein mixture was loaded on an SP Sepharose column (60 ml bed volume). Proteins were separated with a linear gradient of 0 to 100% buffer B (20 mM MES pH 6.8, 1 M NaCl, 1 mM EGTA, 1 mM MgCl2, 2 mM DTT, 0.1 mM PMSF). Fractions were analyzed via SDS-PAGE stained with Coomassie blue. Fractions containing hTau were pooled and dialyzed against 100 volumes of PBS buffer containing 1 mM DTT. The hTau concentration was determined spectrophotometrically using an extinction coefficient at 280 nm of 7450 M−1.cm−1 for Tau 1N3R and 7575 M−1.cm−1 for Tau 1N4R. Aβ1–42 peptides (sequence DAEFRHDSGYEVHHQKLVFFAEDVGSNKGAIIGLMVGGVVIL) were bought from GeneCust (Dudelange, Luxembourg). Aß1–42 was solubilized in HFIP (Sigma, #52517–10 mL) and kept at −20 °C after evaporation of HFIP.

#### Aggregation of protein into amyloid fibrils

α-Syn was incubated in buffer A to obtain the fibrillar polymorph “fibrils” (50 mM Tris–HCl, pH 7.5, 150 mM KCl), in buffer B for “ribbons” (5 mM Tris–HCl, pH 7.5), in buffer C for “fibril-65” (20 mM MES pH6,5, 150 mM NaCl) and in buffer D for “fibril-91” (20 mM KPO4 pH9.1), at 37 °C under continuous shaking in an Eppendorf Thermomixer set at 600 r.p.m for 4–7 days^16,40^.

Fibrillation of human Tau was achieved at 40 μM in the presence of 10 μM heparin by shaking 0.5 ml solution aliquots at 37 °C in an Eppendorf Thermomixer set at 600 rpm for 4 days.

Aß fibrillation was achieved at 500 µM in PBS (Sigma, #D8537) at 37 °C under continuous shaking in an Eppendorf Thermomixer set at 600 r.p.m for 4 days.

#### Labeling of fibrils

All amyloid fibrils were centrifuged twice at 15,000 *g* for 10 min and resuspended twice in PBS at 1.446 g/L. All preformed assemblies were labeled with Atto-550 NHS-ester (Atto-Tec Gmbh # AD 550–35) fluorophore following the manufacturer’s instructions using a protein:dye ratio of 1:2. The labeling reactions were arrested by addition of 1 mM Tris pH 7.5. The unreacted fluorophore was removed by a final cycle of two centrifugations at 15,000 *g* for 10 min and resuspensions of the pellets in PBS.

### Spotting of fibrillar α-Syn, tau and A-beta on different surfaces

Plastic slides (PVC), glass (Glass slides for microscopy, Sigma # S8400-1PAK), aluminum and stainless steel plates (2 × 20 × 60 mm) were pre-treated with sandpaper (grit size P1000) to make the surfaces rougher. 2 µl, containing 1 µg of the different fibrillar assemblies, were spotted on the different surfaces and allowed to dry over night at room temperature in the dark.

### Cleaning procedure

Slides and plates were incubated fully immerged in a petri dish containing 10 ml of washing solution (MilliQ water, SDS (1%), NaOH 1 N, Hellmanex II (1%), TFD4 (1%), guanidine 6 M), under gentle agitation on an orbital shaker (set at 50 rpm) at room temperature for 1 hour. Slides and plates were removed, washed in Petri dishes containing 10 ml of MilliQ under the same agitation conditions. Finally slides and plates were recovered and allowed to dry over night at room temperature in the dark.

### Quantification of assemblies

#### Fluorescence measurements

Fibrils were detected directly on the slides and plates through Atto-550 fluorescence using a ChemiDoc^TM^ MP(BioRad) and the protocol DyLight 549. Acquisition was performed using the protocol ≪ Signal Acquisition Mode ≫ from 0.002 sec to 2 sec of exposition. Images were processed and quantified using Image Lab, briefly, the fluorescence intensity of each spot was integrated using round in ≪ Volume Tool ≫ with a fixed diameter. The background was measured on a neighboring area and subtracted. The proportion of remaining protein was calculated for each spot as the fluorescence intensity after cleaning divided by the initial fluorescence intensity.

#### Inactivation assay

α-Syn, tau and Aß fibrillar assemblies (25 µg) were dried in 500 µl polycarbonate tubes. The different cleaning solutions were added (200 µl) and the tubes were incubated at room temperature for 1 h. The tubes were next subjected to ultracentrifugation (100,000 g for 40 min at 20 °C in a tabletop TL100 ultra centrifuge using a TLA120.1 rotor). The absorbance at 550 nm of the different fibrils diluted in the different cleaning solutions and of the supernatant after centrifugation were recorded using an HP 8453 UV-Vis spectrophotometer (Hewlett Packard).

## Electronic supplementary material

Figure S1, Figure S2
