# Supplementary material for: Assessment of the efficacy of different procedures that remove and disassemble alpha-synuclein, tau and A-beta fibrils from laboratory material and surfaces
Source: Sci Rep. 2018 Jul 17;8:10788. doi: 10.1038/s41598-018-28856-2 (PMC6050226; doi:10.1038/s41598-018-28856-2)
Supplement: Supplementary file 1 — Figure S1, Figure S2 [file 41598_2018_28856_MOESM1_ESM.pdf]

Assessment of the efficacy of different procedures that remove and disassemble alpha-synuclein, tau and A-beta fibrils from laboratory material and surfaces

Supplementary Data

Alexis Fenyi, Audrey Coens, Tracy Bellande, Ronald Melki, Luc Bousset

Paris-Saclay Institute of Neuroscience, Centre National de la Recherche Scientifique, Université Paris-Saclay, 91190 Gif-sur-Yvette, France

Address correspondence to R. Melki, ronald.melki@cnrs.fr; Tel. +33169823503; Fax. +33169823447

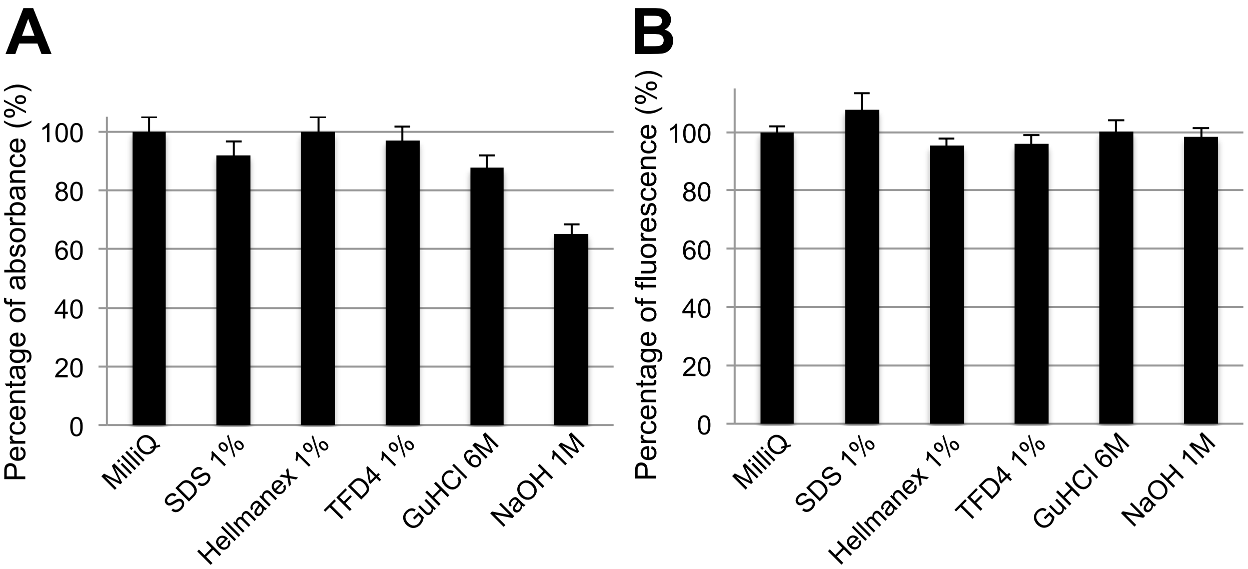

Figure S1. Atto550 absorbance and fluorescence in cleaning solutions. The absorbance (A) of Atto550 (10µM) at 550nm was measured in the different cleaning solutions. The result is adjusted to the absorbance at 680nm and expressed as the fraction of Atto550 absorbance in MilliQ water. The fluorescence (B) of Atto 550 (10µM) at 575nm upon excitation at 555nm in the different cleaning solutions was recorded. The fluorescence is expressed as the fraction of Atto550 fluorescence in MilliQ water.

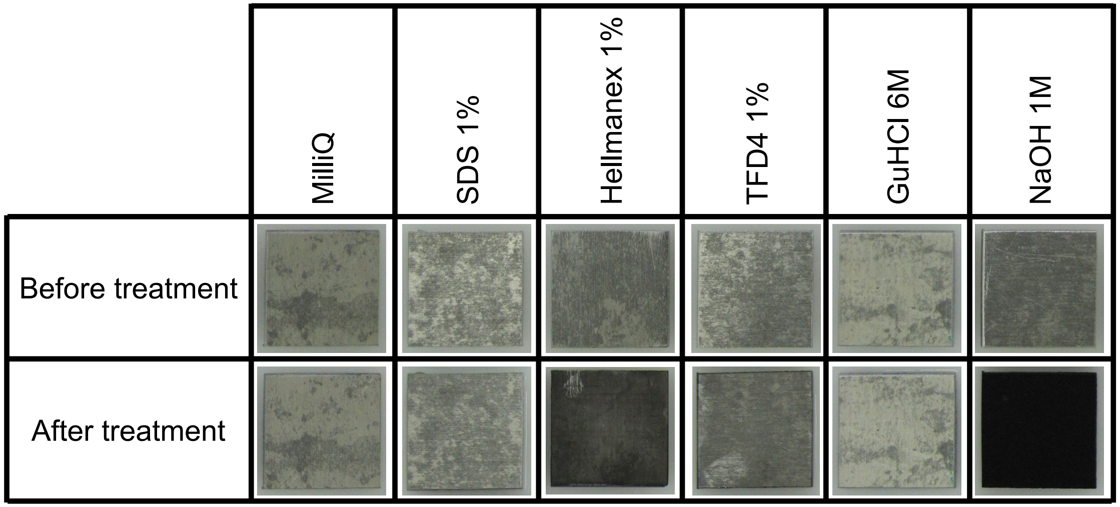

Figure S2. Effect of the different cleaning solutions on aluminum plates. The aluminum plates were photographed before and after been immersion for 1 hour in the indicated cleaning solution under gentle agitation.
